# Clinical Characteristics and Risk Prediction Score in Patients With Mild-to-Moderate Coronavirus Disease 2019 in Japan

**DOI:** 10.7759/cureus.31210

**Published:** 2022-11-07

**Authors:** Atsushi Marumo, Haruka Okabe, Hisae Sugihara, Junichi Aoyama, Yasuhiro Kato, Kensuke Arai, Yasuhiro Shibata, Etsu Fuse, Machiko Nomura, Kiyotaka Kohama

**Affiliations:** 1 Division of Internal Medicine, Fussa Hospital, Tokyo, JPN; 2 Department of Hematology, Nippon Medical School, Tokyo, JPN; 3 Division of Clinical Laboratory, Fussa Hospital, Tokyo, JPN; 4 Department of Pulmonary Medicine and Oncology, Graduate School of Medicine, Nippon Medical School, Tokyo, JPN; 5 Department of Pulmonary Medicine and Oncology, Nippon Medical School, Tokyo, JPN

**Keywords:** risk prediction score, anticoagulants, antibiotic agents, prognostic factor, covid-19

## Abstract

Background: Coronavirus disease 2019 (COVID-19) has rapidly spread worldwide, causing widespread mortality. Many patients with COVID-19 have been treated in homes, hotels, and medium-sized hospitals where doctors were responsible for assessing the need for critical care hospitalization. This study aimed to establish a severity prediction score for critical care triage.

Method: We analyzed the data of 368 patients with mild-to-moderate COVID-19 who had been admitted to Fussa Hospital, Japan, from April 2020 to February 2022. We defined a high-oxygen group as requiring ≥4 l/min of oxygen. Multivariable logistic regression was used to construct a risk prediction score, and the best model was selected using a stepwise selection method.

Results: Multivariable analysis showed that older age (≥70 years), elevated creatine kinase (≥127 U/L), C-reactive protein (≥2.19 mg/dL), and ferritin (≥632.7 ng/mL) levels were independent risk factors associated with the high-oxygen group. Each risk factor was assigned a score ranging from 0 to 4, and we referred to the final overall score as the Fussa score. Patients were classified into two groups, namely, high-risk (total risk factors, ≥2) and low-risk (total risk score, <2) groups. The high-risk group had a significantly worse prognosis (low-risk group, undefined vs. high-risk group, undefined; P< 0.0001).

Conclusions: The Fussa score might help to identify patients with COVID-19 who require critical care hospitalization.

## Introduction

Severe acute respiratory syndrome coronavirus 2 (SARS-CoV-2) was first reported in patients with pneumonia in December 2019 in China, and the disease was subsequently officially named coronavirus disease (COVID-19) by the World Health Organization [1]. COVID-19 rapidly spread worldwide, with high mortality rates, and influenced clinical environments, societies, economies, and lifestyles. No information regarding the characteristics of COVID-19 or its treatment was available initially in Japan or anywhere. Infectious disease specialists mainly treated severe and complicated COVID-19 patients. Because of the shortage of infectious disease specialists, general physicians also treated COVID-19 patients with subsequent confusion in the medical field.

Beigel et al. [2] reported that remdesivir reduced the length of hospital stay in patients with COVID-19 requiring oxygen supplementation. In contrast, Wang et al. [3] reported that remdesivir use did not have a significant effect on recovery. Another study found that early administration of remdesivir lowered the rate of intensive care unit (ICU) admissions and the need for mechanical ventilation in patients with COVID-19 [4]. Several studies from Japan and Russia initially reported that favipiravir shortened the symptom recovery time [5,6]. However, McMahon et al. [7] reported that favipiravir did not improve clinical outcomes. The antiviral drugs remdesivir and favipiravir were used from 2020 to 2021 following the standardization of COVID-19 treatment according to the guidelines for the treatment of COVID-19 in Japan.

Many patients with COVID-19 were treated in their homes, hotels, and medium-sized hospitals (without an ICU). The rapid worsening of 20% of patients with COVID-19 necessitated critical care triage [8]. Initially, some critically ill older patients with COVID-19 voluntarily rejected intensive care treatments such as ventilator treatment and extracorporeal membrane oxygenation. This made a sufficient number of hospital beds and medical resources available to enable the transfer of patients with severe COVID-19 requiring intensive care treatment to critical care hospitals. However, when critical care hospitals experienced bed shortages due to an increase in young patients being admitted with the Delta variant, treating patients with severe COVID-19 in medium-sized hospitals became unavoidable. Some patients with severe COVID-19 died or experienced severe permanent damage because of delayed treatment. A method for better prediction of the prognosis and evaluation of the need for critical care hospitalizations was thus required.

Risk factors associated with disease severity included older age, hypertension, cancer, diabetes mellitus (DM), obesity, cerebrovascular disease, chronic kidney disease, and elevated ferritin, D-dimer, and C-reactive protein (CRP) levels [9-11]. Several prediction scores have been reported with death or ICU admission as the endpoints [9-13]. Death or ICU admission as endpoints for prognostic scores may delay the decision to transfer. Therefore, an earlier stage than death or ICU admission should be considered an endpoint. Several articles have reported that an oxygen demand of 4 l/min is associated with mortality [14,15].

Therefore, we decided to come up with a new prognostic score with an oxygen demand of 4 l/min as the endpoint. This study aimed to investigate the clinical characteristics and treatment results of patients with mild-to-moderate COVID-19 and develop a prediction score.

This article was previously presented as an oral presentation at the 96th Annual Meeting of the Japanese Association for Infectious Disease on April 23, 2022.

## Materials and methods

Setting and patients

**Figure 1:**
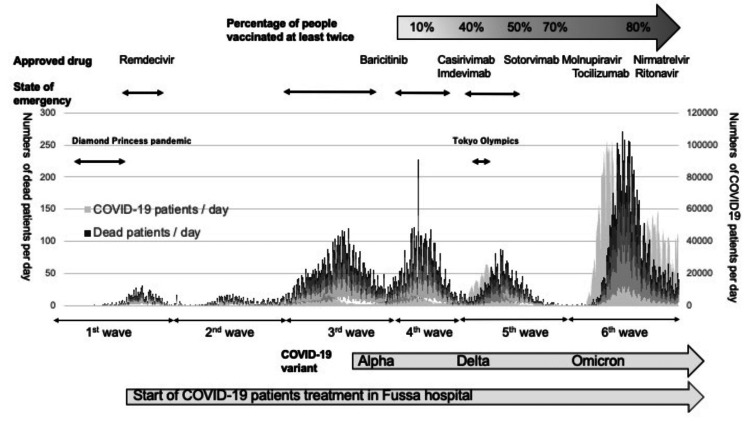
Social conditions and the number of COVID-19 patients in Japan.

This single-center, retrospective, observational study was conducted at Fussa Hospital, Japan. Fussa Hospital is a 316-bed public acute care hospital located in the suburbs of Tokyo. It is not equipped with an ICU, and critically ill patients are transferred to a critical care hospital. A total of 563 patients diagnosed with COVID-19 were admitted to the hospital from April 2020 to February 2022. We defined the first wave of COVID-19 as from January 1 to March 21, 2020; the second wave as from May 26 to October 31, 2020; the third wave as from November 1 to March 21, 2021; the fourth wave as from March 22 to June 20, 2021; the fifth wave as from June 21 to October 31, 2021; and the sixth wave as from December 1, 2021, to January 31, 2022 (Figure 1). We classified COVID-19 patients into four categories at the time of admission according to the National Institutes of Health (NIH) classification criteria [16]. The presence of pneumoniae was assessed using CT or x-ray imaging, and hypoxia was defined as SpO_2_ ≤ 93%. Mild cases were defined as symptomatic patients with no evidence of pneumonia or hypoxia. Moderate cases were defined as patients with pneumonia who did not require oxygen supplementation. Severe cases were defined as patients with pneumonia requiring oxygen supplementation. Critical cases were defined as patients requiring ventilator management. We excluded severe and critical patients. Therefore, we analyzed the treatment and clinical data of 368 patients with mild-to-moderate COVID-19 within three days of hospitalization. The study was conducted in accordance with the principles of the Declaration of Helsinki. Informed consent was waived because all of the data were obtained retrospectively. The data of patients were collected in the study using the opt-out method. The Institutional Review Board of Fussa Hospital approved this study (approval number: 2021-14).

Statistical analyses

In this study, patients requiring ≥4 l/min of oxygen during hospitalization were classified into the high-oxygen group, and those requiring <4 l/min of oxygen or no oxygen were classified into the low- or no-oxygen group. We analyzed the clinical characteristics of these groups using the Mann-Whitney U test for continuous variables and a chi-square test for categorical variables. Statistical significance was defined as a two-sided P-value <0.05. Receiver-operating characteristic (ROC) curves were plotted for continuous variables to determine the cutoff values for predicting demand for ≥4 l/min oxygen. The threshold for the largest area under the ROC curve (AUC) was identified, and the sensitivity and specificity of the cutoff value were then calculated. Predictor variables were selected based on clinical characteristics, comorbidities, symptoms, and biomarkers to construct a risk prediction score, and we included these variables in multivariable logistic regression analysis and selected the ideal model using a stepwise selection method (Bayesian information criterion). Patients were divided into two groups based on the results (low-risk group: total risk score, <2; high-risk group: total risk score, ≥2). Based on these two groups, we calculated event-free survival and used the Kaplan-Meier method to describe the time from the onset of symptoms to requiring ≥4 l/min of oxygen, and the log-rank test was used to compare the two groups. EZR version 1.54 (Saitama Medical Center, Jichi Medical University, Saitama, Japan) and GraphPad Prism (version 9.0 for Mac OS, GraphPad Software, La Jolla, CA) software were used to analyze the clinical characteristics of the two groups [17].

## Results

Characteristics of patients in the high-oxygen and low- or no-oxygen groups

We analyzed 368 patients with mild-to-moderate COVID-19. The median age of the study patients was 52 (range, 1-99) years, and there were slightly more male patients (n = 189, 51.4%). On admission, 136 and 232 patients had mild and moderate COVID-19, respectively. Of the 227 patients for whom vaccination information was available, 64 (28.2%) had been vaccinated. SARS-CoV-2 mutations were detected in 10 patients in the low- or no-oxygen group (L452R in seven, N501Y in one, and unknown in two patients) and in one patient in the high-oxygen group (L452R). Computed tomography and bacterial culture test results showed that seven patients had bacterial pneumonia. Twenty-five patients had been treated with antibiotics. Forty-four patients had been administered anticoagulants, comprising 23, 13, and eight patients treated with heparin, nafamostat mesylate, and direct oral anticoagulants, respectively, as prophylactic anti-thrombosis treatment. A 52-year-old patient with DM, hypertension, and hyperlipidemia had a cerebellar infarction and was not treated with anticoagulants. No patients had anticoagulant-associated hemorrhagic complications during hospitalization.

In the high-oxygen group, patients were significantly older, had more DM, pneumonia, and anorexia, and were infected during the third wave. Laboratory findings on admission showed that the platelet (Plt) count and albumin (Alb) level were significantly lower in the high-oxygen group. In contrast, the levels of aspartate aminotransferase (AST), alanine aminotransferase (ALT), lactate dehydrogenase (LDH), creatine kinase (CK), creatinine (creat), CRP, ferritin, and D-dimer were significantly elevated. Corticosteroids, favipiravir, and antibiotics were used more frequently in the high-oxygen group (Table 1).

**Table 1:** Patient characteristics of the high-oxygen group and low-oxygen group. *We collected vaccination information from 227 patients (211 patients in the non-critical group and 16 patients in the critical group). **We collected smoking information from 358 patients (339 patients in the non-critical group and 19 patients in the critical group). ***WBC, Plt, AST, ALT, BUN, and Creat were examined in 349 patients. Lymphocyte, Alb, LDH, T-bil, CK, CRP, ferritin, D-dimer, and KL-6 were examined in 305, 340, 344, 346, 316, 348, 255, 310, and 98 patients, respectively. Bold values represent the P value was less than 0.05. Alb, albumin; ALT, alanine aminotransferase; AST, aspartate aminotransferase; BMI, body mass index; BUN, blood urea nitrogen; CK, creatine kinase; CKD, chronic kidney disease; Creat, creatinine; CRP, C-reactive protein; COPD, chronic obstructive pulmonary disease; KL-6, Krebs von den Lungen-6; LDH, lactate dehydrogenase; Plt, platelet; T-bil, total bilirubin; WBC, white blood cell.

	Low- or no-oxygen (n = 348)	High-oxygen (n = 20)	P-value
Age, median years old (range)	50.0 (1.0-99.0)	79.5 (31.0-96.0)	<0.001
Sex (male) (%)	175 (50.3)	14 (70.0)	0.108
BMI, median kg/m^2^ (range)	23.1 (14.9-42.7)	23.1 (15.2-36.1)	0.792
Coronavirus vaccination*	62 (29.3)	2 (10.0)	0.247
Smoking history (%)**	138 (40.7)	6 (31.6)	0.481
Hypertension (%)	91 (26.1)	8 (40.0)	0.196
Diabetes (%)	38 (10.9)	6 (30.0)	0.022
COPD (%)	6 (1.7)	1 (5.0)	0.363
Cancer (%)	20 (5.7)	0 (0.0)	1
Hyperlipidemia (%)	40 (11.5)	3 (15.0)	0.717
CKD (%)	5 (1.4)	1 (5.0)	0.287
Period at diagnosis (%)			
First wave	9 (2.6)	0 (0.0)	1
Second wave	27 (7.8)	1 (5.0)	1
Third wave	57 (16.4)	9 (45.0)	0.004
Fourth wave	53 (15.2)	3 (15.0)	1
Fifth wave	132 (37.9)	5 (25.0)	0.342
Sixth wave	70 (20.1)	2 (10.0)	0.388
Symptoms on admission			
Fever, median ℃ (range)	37.2 (35.8-40.1)	37.8 (35.6-39.3)	0.167
Cough	171 (49.1)	12 (60.0)	0.368
Sputum	42 (12.1)	2 (10.0)	1
Runny nose	24 (6.9)	3 (15.0)	0.173
Sore throat	56 (16.1)	0 (0.0)	0.054
Dyspnea	62 (17.8)	7 (35.0)	0.073
Dizzy	5 (1.4)	0 (0.0)	1
Fatigue	96 (27.6)	7 (35.0)	0.453
Nausea	30 (8.6)	1 (5.0)	1
Diarrhea	26 (7.5)	1 (5.0)	1
Headache	68 (19.5)	1 (5.0)	0.142
Chest pain	14 (4.0)	0 (0.0)	1
Abdominal pain	5 (1.4)	0 (0.0)	1
Olfactory disorder	20 (5.4)	0 (0.0)	0.614
Taste disorder	20 (5.4)	0 (0.0)	0.614
Anorexia	13 (3.7)	3 (15.0)	0.049
Muscle or joint pain	33 (9.5)	0 (0.0)	0.238
Chilling	2 (0.6)	1 (5.0)	0.155
Laboratory data at diagnosis***			
WBC, median × 10^2^/μL (range)	48.0 (19.0-205.0)	52.0 (22.0-113.0)	0.147
Lymphocyte, median × 10^2^/μL (range)	1,085.5 (315.0-3705.0)	881.3 (234.0-2,264.9)	0.191
Plt, median × 10^4^/μL (range)	18.9 (7.6-40.1)	14.8 (5.20-31.7)	<0.001
Alb, median g/dl (range)	4.00 (2.00-5.00)	3.60 (2.70-4.60)	0.001
T-bil, median mg/dl (range)	0.51 (0.17-2.03)	0.44 (0.30-1.48)	0.848
AST, median IU/L (range)	27.0 (12.0-254.0)	50.0 (16.0-144.0)	<0.001
ALT, median IU/L (range)	22.0 (3.0-246.0)	35.0 (13.0-170.0)	0.049
LDH, median IU/L (range)	207.0 (106.0-524.0)	257.0 (160.0-357.0)	0.002
CK, median IU/L (range)	79.0 (20.0-8688.0)	132.0 (22.0-615.0)	0.024
BUN, median mg/dl (range)	12.1 (4.4-96.6)	13.6 (6.4-28.6)	0.089
Creat, median mg/dl (range)	0.8 (0.27-5.9)	0.9 (0.53-1.50)	0.075
CRP, mg/dL (range)	1.4 (0.01-20.7)	4.8 (0.6-17.0)	<0.001
Ferritin, ng/ml (range)	219.9 (0.4-4019.0)	390.6 (71.6-1517.7)	0.005
KL-6, U/ml (range)	222.0 (97.0-1290.0)	181.0 (153.0-191.0)	0.170
D-dimer, μg/ml (range)	0.90 (0.50-96.8)	1.00 (0.70-17.40)	0.006
Treatment (%)			
Corticosteroid	56 (16.2)	15 (75.0)	<0.001
Favipiravir	12 (3.4)	6 (30.0)	<0.001
Remdesivir	131 (37.6)	8 (40.0)	0.817
Baricitinib	5 (1.4)	0 (0.0)	1
Sotrovimab	17 (1.0)	0 (0.0)	0.613
Molnupiravir	4 (1.1)	0 (0.0)	1
Anticoagulant	39 (11.2)	5 (25.0)	0.076
Antibiotics	21 (5.6)	4 (16.7)	0.038
Period from diagnosis to administration, median days (range)	4 (0-33)	4 (0-9)	0.577
Hospitalization period, median days (range)	9 (0-50)	12 (1-39)	0.694
Oxygen administration period, median days (range)	0 (0-32)	0 (0-28)	0.695
Presence of pneumonia (%)	213 (61.2)	19 (95.0)	0.001

Establishing the risk prediction score and event-free survival based on the Fussa score

We plotted ROC curves with the continuous variables, calculated their cutoff values (Table 2), and created two groups based on these cutoff values. The univariate analysis revealed 14 significant variables (age, plt count, alb, AST, ALT, LDH, CK, CRP, ferritin, and D-dimer levels, DM, anorexia, presence of pneumonia, and third wave), which were included in the multivariable logistic regression analysis. Multivariable analysis revealed that older age (≥70 years) and elevated CK (≥127 U/L), CRP (≥2.19 mg/dL), and ferritin (≥632.7 ng/mL) levels were independent risk factors associated with an oxygen demand of ≥4 l/min (Table 3). The presence of each risk factor was assigned one point (scores ranging from 0 to 4), and the final overall score was referred to as the Fussa hospital score. The ROC curve of the Fussa hospital score showed that the AUC was 0.879, and the most efficient cutoff point was 2.0 (Table 2). 

**Table 2:** ROC curves at age and for each biomarker. Alb, albumin; ALT, alanine aminotransferase; AST, aspartate aminotransferase; AUC, area under the curve; CK, creatine kinase; CRP, C-reactive protein; LDH, lactate dehydrogenase; Plt, platelet; ROC curve, receiver-operating characteristics curves. We drew the ROC curve of age, Plt, Alb, AST, ALT, LDH, CK, CRP, ferritin, D-dimer, and Fussa hospital score. We also calculated AUC, cutoff value, sensitivity, and specificity to the oxygen demand of 4 l/min with the Youden index.

	Cutoff	Sensitivity (%)	Specificity (%)	AUC
Age (n = 368)	70.00	70.0	72.7	0.740
Plt (n = 349)	15.40	75.0	75.0	0.740
Alb (n = 340)	3.80	85.0	57.5	0.725
AST (n = 349)	38.00	70.0	75.1	0.758
ALT (n = 349)	35.00	55.0	69.6	0.631
LDH (n = 344)	223.00	78.9	62.2	0.712
CK (n = 316)	127.00	57.9	76.8	0.655
CRP (n = 348)	2.19	85.0	59.5	0.753
Ferritin (n= 255)	632.70	46.7	88.7	0.715
D-dimer (n =310)	0.90	89.5	46.4	0.693
Fussa hospital score (n = 368)	2.00	95.0	73.3	0.879

**Table 3:** Multivariate analysis. CK, creatine kinase; CRP, C-reactive protein. Bold values represent the P value was less than 0.05. We performed multivariable logistic regression analysis using the variables from univariate analysis and selected the ideal model using a stepwise selection method.

	P value	Odds ratio	95% Confidence interval
CK ≧ 127 IU/L	0.012	5.35	1.45-19.70
CRP ≧ 2.19 mg/dL	0.032	6.10	1.17-31.70
Ferritin ≧ 632.7 ng/mL	0.003	9.14	2.15-38.80
Age ≧ 70 years old	0.010	6.44	1.57-26.40

**Figure 2:**
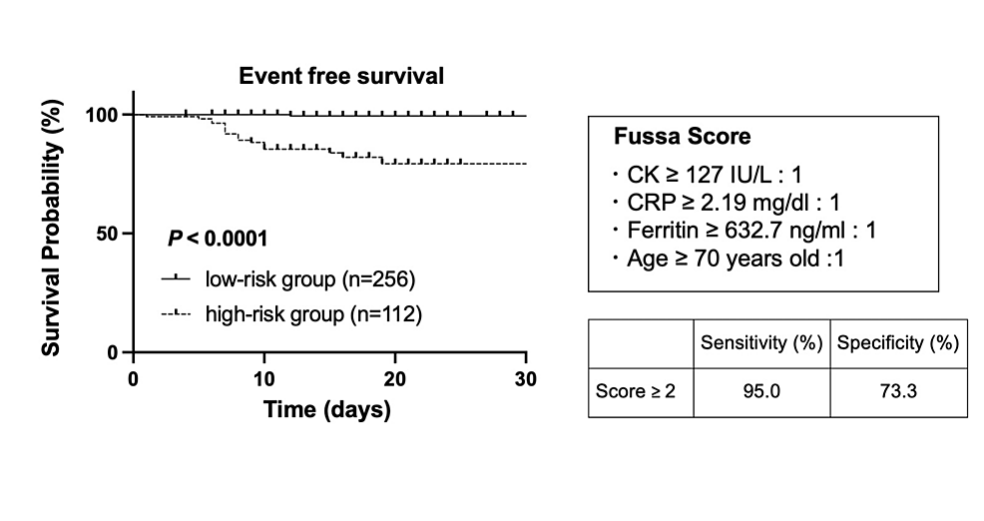
Event-free survival based on the Fussa hospital score. High-risk group: score, ≥2 (n = 89); low-risk group: score, <2 (n = 232). According to Table 2, we analyzed the cutoff value of the Fussa hospital score and classified two groups. We defined the endpoint as the demand for ≥4 l/min oxygen.

We classified the patients into two groups based on the Fussa score: high-risk (total risk factors, ≥2) and low-risk (total risk score, <2). The event-free survival was calculated for each group, and the demand for ≥4 l/min oxygen was significantly associated with the high-risk group (low-risk group, undefined vs. high-risk group, undefined; P < 0.0001) (Figure 2).

## Discussion

In this study, we analyzed 368 patients with mild-to-moderate COVID-19. Of these, 25 (6.8%) patients had been prescribed antibiotic agents; however, only seven (1.9%) patients with bacterial pneumonia had required antibiotic treatment. The rate of bacterial co-infection was relatively low in this study as compared with that reported in another study (3.5%) [18] since we excluded critically ill patients from the analysis. The antibiotic prescription should be limited because antibiotics might increase fatality rates and organ damage [19].

A 52-year-old patient with moderate COVID-19 had a cerebellar infarction, and he was administered anticoagulants for prophylaxis. We usually only administer anticoagulant prophylaxis to patients with severe or critical COVID-19 because only 0.59% of patients with mild-to-moderate COVID-19 have been reported to present with thrombosis [20]. However, because thrombosis sometimes occurs even in patients with mild-moderate COVID-19, we should pay attention to the patients, especially those who are at high risk of thrombosis, such as those with DM.

In previous reports, old age, DM, low plt count, and alb level, and high AST, ALT, LDH, CK, Creat, CRP, ferritin, and D-dimer levels were found to be poor prognostic factors [9-11]. We defined the Fussa score (age, ≥70 years; CK, ≥127 U/L; CRP, ≥2.19 mg/dL; and ferritin, ≥632.7 ng/mL) based on our multivariable analysis. Previous studies have also established prediction scores; however, these prediction scores could not be readily used for the transfer of patients to critical care hospitals as their endpoints were death or ICU admission [9-13]. The endpoint of the Fussa score was the requirement for ≥4 l/min of oxygen, and the Fussa score might be useful for evaluating the need for critical care hospitalizations. The Fussa score has low specificity (73.3%) and high sensitivity (95.0%) and excludes patients requiring ≥4 l/min of oxygen.

Our study had several limitations. First, this was a single-center study, and the sample size was small. We could not perform a validation cohort study to confirm the effectiveness of the risk prediction score, and overfitting was a possibility because of the small sample size. Our sample size was not sufficiently large to conduct a precise multivariable analysis. Second, we were not able to collect enough data related to SARS-CoV-2 mutation and vaccination to analyze their influence on prognosis adequately. Instead, we collected information concerning vaccination status and prevalent mutant strains at each time period from the first wave to the sixth wave. Finally, our institution is a medium-sized hospital, and we could not perform intensive care management. We transferred critical patients to critical care hospitals and could not follow the clinical data until the end of patient treatment.

## Conclusions

In this study, we created the Fussa hospital score (age, ≥70 years; CK, ≥127 U/L; CRP, ≥2.19 mg/dL; and ferritin, ≥632.7 ng/mL). The patients with mild-to-moderate COVID-19 and a Fussa hospital score ≥2 points were significantly susceptible to requiring ≥4 l/min oxygen. These variables are available and easily accessible to most hospitals and clinics.

The Fussa hospital score might be useful for doctors treating patients with mild-to-moderate COVID-19 in medium-sized hospitals or homes when assessing the need for critical care hospitalizations.

Validating these scores prospectively to predict the need for critical care hospitalization in other cohorts will establish the usefulness of this scoring system. Clinicians should pay attention to the Fussa hospital score and intervene early.
